# Seroepidemiology of *Chlamydia trachomatis* Infection in the General Population of Northern China: The Jidong Community Cohort Study

**DOI:** 10.3389/fmicb.2021.729016

**Published:** 2021-09-27

**Authors:** Jingwei Shui, Dongjie Xie, Jianhui Zhao, Cailing Ao, Hongqing Lin, Yuanhao Liang, Haiying Wang, Yingchun Dai, Shixing Tang

**Affiliations:** ^1^Department of Epidemiology, School of Public Health, Southern Medical University, Guangzhou, China; ^2^Wenzhou Institute, University of Chinese Academy of Sciences, Wenzhou, China

**Keywords:** *Chlamydia trachomatis*, anti-Pgp3 antibody, general population, incidence, antibody decay, seropositive frequency

## Abstract

A longitudinal serological study to investigate the seropositive frequency, incidence, and antibody dynamics of *Chlamydia trachomatis* infection in the general population of China is urgently needed in order to optimize the strategies for surveillance and precise prevention of *C. trachomatis* infection. This longitudinal study enrolled 744 subjects aged 18–65 years from Jidong Community of Northern China from 2014 to 2018. Seropositive frequency, incidence, and reinfection of *C. trachomatis* were determined by detecting antibody against *C. trachomatis* Pgp3 using “in-house” luciferase immunosorbent assay (LISA). The dynamic of anti-Pgp3 antibody was analyzed using the Generalized Estimating Equation (GEE) model. The overall Pgp3 seropositive frequency among the 18–65-year-old population was 28.1% (95% CI 24.9–31.5), and significantly increased from 12.0% in those aged 18–29 years to 48.6% in the 60–65 years old. The seropositive frequency was slightly higher in women than in men (31.3% vs. 25.4%) without statistical significance. The *C. trachomatis* incidence and reinfection rate were 11 and 14 per 1,000 person-years, respectively, and showed no significant difference with respect to age, gender, ethnicity, marital status, and education levels. Furthermore, anti-Pgp3 antibody remained detectable in 93.3% (195/209) of the seropositive subjects during the 5 years of follow-up. The overall decay rate for anti-Pgp3 antibody for CT-infected persons was −0.123 Log2 RLU/year, which was dramatically slower than in CT new infection (−3.34 Log2 RLU/year) or reinfection (−1.1 Log2 RLU/year). In conclusion, at least one quarter of the people aged 18–65 years have been infected with *C. trachomatis* over their lifetime while all age groups are susceptible to *C. trachomatis* infection in the community of Northern China. Therefore, comprehensive prevention strategies are urgently needed.

## Introduction

Genital *chlamydia trachomatis* (CT) infection is the most common, curable sexually transmitted disease (STD) worldwide (Woodhall et al., 2018). As up to 80% of cases are asymptomatic, CT infection often remains undetected or not diagnosed, which in turn results in the wide spread and delayed treatment of CT infection (Marcone et al., 2012). Furthermore, untreated CT infection can cause scarring of the upper reproductive tract in women and lead to serious complications, such as pelvic inflammatory disease, ectopic pregnancy, and tubal factor infertility (Haggerty et al., 2010). In men, CT infection is the most common cause of non-gonococcal urethritis and accessory gland infection (Wagenlehner et al., 2006). CT infection is also reported to be associated with increased risk of cervical cancer and acquisition of human immunodeficiency virus (HIV) infection (Koskela et al., 2000; Anttila et al., 2001; Petersen et al., 2020).

*Chlamydia trachomatis* infection is increasing worldwide from about 89 million new cases in 1995 to 127.2 million in 2016 (Newman et al., 2015; Rowley et al., 2019). In 2016, based on nucleic acid testing data, the global prevalence of *C. trachomatis* was estimated to be 3.8% in 15–49-year-old women and 2.7% in men whereas the global incidence rate was 34 cases per 1,000 person-years in women and 33 per 1,000 person-years in men, respectively (Rowley et al., 2019). In 2018, the European Centre for Disease Prevention and Control (ECDC) reported a crude *C. trachomatis* prevalence of 0.15% in 26 EU/EEA countries, and 5.3% increase of the prevalence from 2009 to 2018.^1^ In the United States, there were more than 1.7 million *C. trachomatis* infections in 2018 (Torrone et al., 2014).

Given the significant burden of *C. trachomatis* infection and the serious sequelae, national *C. trachomatis* screening program has been implemented mainly targeting young women under 25 years old in several countries including England, Netherlands, and United States (Marcone et al., 2012; Workowski and Bolan, 2015) because they usually compose the majority of the reported *C. trachomatis* new infections (Fine et al., 2012). In these countries, a substantial decrease of CT infection-related complications has been observed at the end of the 1980s and early 1990s and a further decrease of pelvic inflammatory disease and ectopic pregnancy has been documented since 2000 (Chandra et al., 2005; Sutton et al., 2005; Bender et al., 2011; French et al., 2011; Marcone et al., 2012).

Unfortunately, there is no nationwide screening program of CT infection in China where bacterial sexually transmitted infections (STIs) are highly prevalent and have rapidly spread since early 1980s (Chen et al., 2000, 2011). Chlamydia is not a notifiable infectious disease in China although it has been included as a reportable STI in the national STI surveillance program since 2008 (Yue et al., 2020). Available data on the prevalence of CT infection are limited and a few prevalence surveys specifically targeted *ad hoc* populations, such as female sex workers (FSWs), men who have sex with men (MSM), immigrants, clinical patients, or pregnant women (Chen et al., 2011). A population-based study conducted in 1999 to 2000 that enrolled 3,426 Chinese residents using nucleic acid test showed a prevalence of 2.1% in men and 2.6% in women (Parish et al., 2003). In addition, several studies indicated that the CT prevalence ranged from 1.5 to 5.4% in the general population of women (Xia et al., 2015; Zhang et al., 2017; Rowley et al., 2019). So far, there is no longitudinal study about *C. trachomatis* infection in China.

Previous studies usually adapted nucleic acid amplification test (NAAT) to detect current *C. trachomatis* infection, rather than past infection. It is resource intensive and impossible to conduct population-based screening of *C. trachomatis* infection by using NAAT (Fine et al., 2012). In contrast, serological assays can determine the prevalence of both current and past *C. trachomatis* infection (Horner, 2017) and have been successfully used in several countries to assess *C. trachomatis* epidemiology (Lyytikäinen et al., 2008a; Horner et al., 2013, 2016; van Aar et al., 2014; Righarts et al., 2017; Woodhall et al., 2017). The performance of serological testing for *C. trachomatis* has been dramatically improved due to the identification of plasmid gene product 3 (Pgp3) antigen to avoid cross-reactivity with other *Chlamydia* spp. (Wills et al., 2009) and to improve suboptimal sensitivity (Woodhall et al., 2018). Immunoglobulin G (IgG) antibody against Pgp3 has been found to be the most reliable marker of *C. trachomatis* infection because Pgp3 is highly conserved across isolates and rarely found in *C. pneumoniae* (Gwyn et al., 2017; Kaur et al., 2018). Serological assays based on *C. trachomatis* Pgp3 antigen also outperformed other *C. trachomatis* antigens (Horner et al., 2016).

*Chlamydia trachomatis* serological assay provides a tool to investigate the prevalence and incidence of *C. trachomatis* infection. A thorough investigation of *C. trachomatis* infection including prevalence and incidence in general population is crucial to identify unmet needs for precise prevention and treatment services (Woodhall et al., 2018), especially in China where there is huge population size but no population-based survey of *C. trachomatis* infection. In this study, we explored the seropositive frequency and incidence of CT infection in the general population aged 18–65 years in a longitudinal community cohort from 2014 to 2018 in Northern China to achieve deep insight into the CT epidemic and anti-Pgp3 antibody dynamics in China.

## Materials and Methods

### Study Design and Participants of the Jidong Cohort

The Jidong Community Cohort Study of China is a community-based longitudinal study to investigate how the suboptimal health conditions contribute to the incidence and progress of non-communicable chronic diseases among Chinese adults (Wang et al., 2016). The Jidong community is composed of the oil field employees and their family members. The Jidong oil field affiliated hospital provided them annual health examination for free. In 2014, there were a total of 10,043 residents aged over 18 years in the community, and they were all included in the health examination plan and invited to participate the cohort study of non-communicable chronic diseases through a convenience sampling. Eventually, 90.4% (9,078/10,043) of the residents attended health examination and completed the questionnaire in the hospital, whereas 965 residents refused to answer the questionnaire or to offer serum samples. Participants were interviewed with a combination of computer-assisted face-to-face and self-completion questionnaires. Sociodemographic data including age, sex, race/ethnicity, marital status, and education were collected when the participants visited the hospital for annual physical examination. Marital status was categorized as either “unmarried,” “married,” and “widowed/divorced/separated.” The serum samples were also collected annually and kept at −80°C until analysis.

**FIGURE 1:**
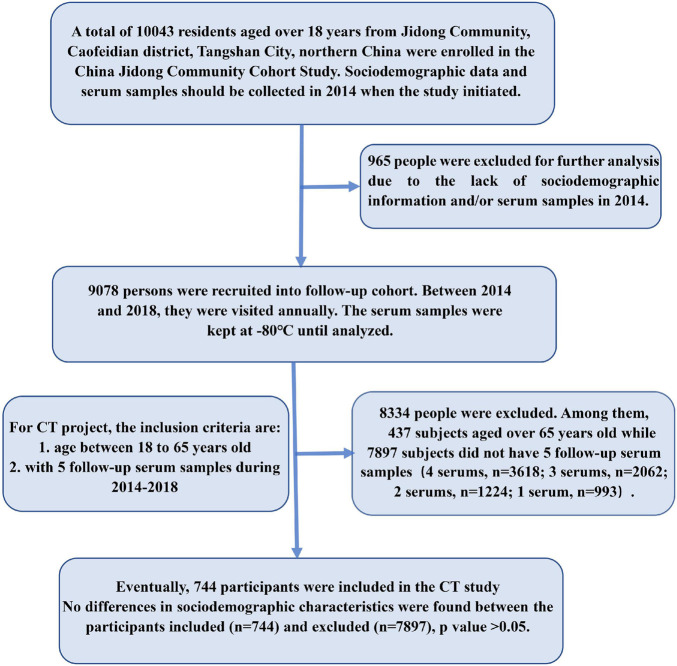
Flowchart of participant enrollment.

For our *C. trachomatis* study, we retrospectively selected the subjects as a longitudinal cohort from the participants aged 18–65 years with five annual health examination records and serum samples from 2014 to 2018 to investigate the seroepidemiology of *C. trachomatis* infection and the dynamics of antibody against *C. trachomatis* Pgp3 protein. The sample size was estimated to be 616 based on the CT seroprevalence of 21.7% in the general population of the United Kingdom according to the Health Survey for England Study (HSE2010/2012) by using the equation: [n=(Zα   2)⁢P⁢(1-P)/d2] in which *P* = 21.7%, α = 0.05, *Z*_α_ = 1.96, allowable error *d* = 0.15*P*. Eventually, a total of 744 participants were included in our study with the following inclusion criteria: (1) age between 18 and 65 years old, and (2) with five continuous annual follow-up serum samples (Figure 1). Although it was not a random sampling, the subjects included and excluded in the current study showed no significant differences with respect to age, gender, ethnicity, marital status, and education levels (*p* > 0.05, Supplementary Table 1). This study was approved by the Ethical Committee of the Jidong Oil Field Hospital of China National Petroleum Corporation (Project No. 2013yilunzi1). Written informed consent was obtained from all the participants.

### *Chlamydia trachomatis* Pgp3-Based Luciferase Immunosorbent Assay

We have previously described an ultrasensitive and high-throughput luciferase immunosorbent assay (LISA) for detection of anti-HIV-1 antibody, which was 10^4^-fold more sensitive than enzyme-linked immunosorbent assay (ELISA) (Wang et al., 2019). The LISA system has further been used to detect antibody against Zika virus and SARS-CoV-2 (Wang et al., 2020; Liang et al., 2021). In this study, we adapted the “in-house” LISA to develop the assay for detection of IgG antibody against the *C. trachomatis*-specific antigen Pgp3.

In brief, the full *C. trachomatis* Pgp3 gene was amplified from a patient infected with *C. trachomatis* serovar E and further confirmed as 100% identity with the *C. trachomatis* strain E-103 plasmid CtrE-103 (accession number: CP015295.1). The Pgp3 gene was then subcloned into the pNLF1-N luciferase expression vector (Promega, Madison, WI, United States) downstream of the Nluc luciferase gene. The recombinant plasmid was transfected into HEK-293T cells (ATCC CRL-3216) and the cell lysates containing Nluc-Pgp3 fusion protein expressed in HEK-293T cells were harvested and confirmed using anti-luciferase antibody. The expressed Nluc-Pgp3 lysates were stored at −80°C until use. The white microtiter plates (Corning, NY, United States) were coated with Protein G (5 μg/ml, 50 μl/well; Genscript, Nanjing, China) or monoclonal mouse anti-human IgG3 (5 μg/ml, 50 μl/well; Eastmo Biotech, Beijing, China), respectively, in 0.01 M PBS and incubated overnight at 4°C. The plates were then washed three times with 0.01 M PBS containing 0.05% Tween 20 (PBS-T) and blocked with 5% non-fat dry milk (NFDM) for 1 h at 37°C. After three washes with PBS-T, 50 μl diluted sera (1:100 dilution in 2% NFDM) were added to the wells and incubated for 1 h at 37°C, followed by five washes with PBS-T. Then Nluc-Pgp3 lysates were added in 50 μl of 2% NFDM (equal to 5 × 10^5^ LU) and incubated at 37°C for 30 min. After five washes with PBS-T, 50 μl of the Nano-Glo Luciferase assay reagent was added to each well to determine the LU according to the manufacturer’s protocol. The average luciferase light units (LU) of triplicate testing for each sample were recorded as crude LU and divided by the average LU of negative controls to get relative light units (RLUs) presented as Log2 RLU. To evaluate the performance of our in-house LISA, we used serum samples from 125 women attending the clinics of Guangzhou Dermatology Hospital and the First People’s Hospital of Chenzhou. All the patients had been diagnosed as *C. trachomatis* current infection by NAAT test. The negative control sera were from 125 healthy, low-risk children aged between 1 and 6 years and stored at the School of Public Health, Southern Medical University, China. These children were assumed not to expose to *C. trachomatis*. Written consent was from all serum donors or their parents. The cut-off value of anti-Pgp3 IgG LISA was determined according to the receiver operating characteristic curve (ROC) on 125 low-risk children’s samples and 125 samples from *C. trachomatis* NAAT positive women. The cut-off value of anti-Pgp3 IgG3 LISA was derived from the average value of the 125 negative controls plus 3 standard deviations (SD). In addition, the performance of Pgp3-based LISA was compared to a commercial recomWell *C. trachomatis* IgG ELISA kit from Mikrogen (Mikrogen GmbH, Neuried, Germany). The kit can detect antibodies against MOMP, translocated actin-recruiting phosphoprotein (TARP) and chlamydial protease-like activity factor (CPAF) (van Ess et al., 2017). The antibodies from *C. trachomatis*-positive and -negative control sera were detected and the cut-off values were determined according to the manufacturers’ instructions.

The positive likelihood ratio (+LR) is defined as the ratio between the probability of positive test in subjects with the disease compared to those without disease (+LR = sensitivity/1–specificity), whereas the negative likelihood ratio (−LR) is the ratio of the probability that a negative result would occur in subjects with the disease so that the same result would occur in subjects without the disease (−LR = 1–sensitivity/specificity). Assays with +LR ≥ 10 and −LR ≤ 0.1 are considered to provide strong evidence to rule in or rule out diagnoses, respectively, in most circumstances (Rahman et al., 2018a). Positive predictive value (PPV) is defined as the probability that the disease is present when the test is positive, whereas negative predictive value (NPV) is defined as the probability that the disease is absent when the test is negative, with formulas as follows: PPV = [sensitivity [×] prevalence] [÷][(sensitivity [×] prevalence) + (1–specificity) [×] (1–prevalence)] and NPV = [specificity [×] (1–prevalence)] [÷] [(1–sensitivity) [×] prevalence + specificity [×] (1–prevalence)] (Maxim et al., 2014). The predictive values (PPV and NPV) were calculated at the antibody frequency observed in this cohort study. Assays with both PPV and NPV exceeding 90.9% are considered high-performance (Rahman et al., 2018a).

### Definition of *C. trachomatis* Infection

Pgp3 is specific for *C. trachomatis* and has been found to be highly immunogenic in its native trimeric form (Wang et al., 2010). According to the previous reports, immunoglobulin G (IgG) is the predominant serum anti-CT antibody in the CT-infected patients (Bakshi et al., 2017). Pgp3 has thus been widely used as a specific and sensitive biomarker of CT infection in the previous studies (Lyytikäinen et al., 2008a; Horner et al., 2013, 2016; van Aar et al., 2014; Righarts et al., 2017; Woodhall et al., 2017) and in the current study. The positive rate of anti-Pgp3 IgG represents the prevalence or frequency of CT infection (Horner et al., 2016). Based on the anti-Pgp3 IgG serostatus, we further defined CT new infection as seroconversion from anti-Pgp3-negative subjects to anti-Pgp3 IgG-positive, while CT reinfection was determined when the titers of anti-Pgp3 IgG increased by at least fourfold among the subjects positive for anti-Pgp3 IgG. To calculate CT incidence or reinfection rate, the person-years for the CT incidence consisted of the total person-years from persistent seronegative subjects during 2014 to 2018 and the person-years observed before the seroconversion of new infected subjects while the person-years for the CT reinfections rate contained the total person-years from initial anti-Pgp3-positive individuals in 2014 and those observed after the seroconversion of new infected subjects. Then, CT incidence is determined by dividing the number of CT new infected individuals over the person-years observed during the study period of 2014 and 2018. Similarly, CT the reinfection rate is calculated by dividing the number of CT reinfected cases over the person-years observed.

### Statistical Analysis

Statistical analysis was done using SPSS 25.0 software (IBM). The *p* value was calculated using chi-square tests (Pearson chi-square, continuity correction, and Fisher’s exact test) for categorical variables and the Mann–Whitney *U*-test for non-categorical variables. Receiver operating characteristic (ROC) analysis was performed to calculate the area under-ROC curves (AUCs) and sensitivities with 95% binominal exact confidence interval (CI). Delong test was used to compare ROC curves. McNemar tests were used to compare the sensitivities between assays; 95% “exact” Clopper–Pearson confidence intervals of the seropositive frequency, incidence, and reinfection rate were estimated by the MedCalc software. Poisson regression was used to analyze the risk factors associated with *C. trachomatis* seropositive frequency using both univariable and multivariable models. The decay rate of anti-Pgp3 antibody levels was estimated using the GEE model implemented in the Stata software. Empirical *p* value was used to show the significance of difference in the antibody decay rates and calculated by the Fisher’s permutation test in Stata software. Statistical significance was set at *p* < 0.05.

## Results

### Evaluation of LISA for Anti-Pgp3 Antibody Detection

Receiver operating characteristic curve analysis showed that the Pgp3-based LISA produced comparable AUC with the commercial Mikrogen ELISA (0.986 vs. 0.993, *p* = 0.209; Table 1). Pgp3-based LISA acquired the maximum Youden index of 0.928 and presented a sensitivity of 92.8% and specificity of 100% at the cut-off value of Log2 RLU = 4.1 (Table 1). Our LISA assay and the Mikrogen ELISA showed similar sensitivity (92.8% vs. 93.6%) and 100% of specificity when detecting the same panel of samples (Table 1). Additionally, the two assays showed 100% of positive predictive value and similar NPV (97.2% for Pgp3-based LISA and 97.5% for Mikrogen ELISA, Table 1). Both the positive likelihood ratio (+LR) and negative likelihood ratio (−LR) indicate the high performance of Pgp3-based LISA (+LR = infinite, −LR = 0.072) and Mikrogen ELISA (+LR = infinite, −LR = 0.064) in detecting anti-*C. trachomatis* IgG antibody.

**TABLE 1:** Performance of Pgp3-based LISA and the commercial Mikrogen ELISA when testing *C. trachomatis*-positive and negative sera*.

Characteristic (95% CI)	Pgp3-based LISA	Mikrogen ELISA	*P* value^#^	High performance range
Area under the ROC curve (AUC-ROC)	0.986 (0.963–0.997)	0.993 (0.973–0.999)	0.209	0.9–1
Sensitivity	92.8% (86.8–96.7%)	93.6% (87.8–97.2%)	>0.999	–
Specificity	100% (97.1–100%)	100% (97.1–100%)	>0.999	–
Positive likelihood ratio^∮^ (+LR)	infinite	infinite	–	≥10
Negative likelihood ratio (−LR)	0.072 (0.04–0.1)	0.064 (0.03–0.1)	–	≤0.1
Positive predictive value^∫^ (PPV)	100%	100%	–	≥90.9%
Negative predictive value (NPV)	97.2% (94.9–98.5%)	97.5% (95.2–98.7%)	–	≥90.9%

*In our study, 125 *C. trachomatis* NAAT-positive and 125 low-risk children’s sera were used to evaluate the performance of Pgp3-based LISA and the commercial Mikrogen ELISA.

*^#^Comparisons of sensitivity and specificity between assays used methods for paired proportions two-sided McNemar’s test. Comparisons of AUC between assays used Delong test.*

*^∮^The positive likelihood ratio tends to be infinite because of the 100% specificity according to the formula “+LR = sensitivity/1–specificity.”*

*^∫^The predictive values were calculated at the 28.1% antibody frequency observed in this cohort study.*

Furthermore, among the 125 *C. trachomatis* NAAT positive sera, the serovars of *C. trachomatis* were determined in 31 samples by ompA sequencing, including 11 serovar F, 6 serovar E, 5 serovar D, 5 serovar J, 2 serovar H, and 2 serovar K (Supplementary Figure 1). Except for one sample with serovar E, the sera with known serovars of *C. trachomatis* were positive for anti-Pgp3 IgG antibody by our Pgp3-based LISA and showed no significantly different antibody titers among the different serovars (*P* = 0.675, Supplementary Figure 1).

### Seropositive Frequency of and Risk Factors Associated With *C. trachomatis* Infection

A total of 3,720 serum samples from 744 subjects were analyzed in this study. The majority of the participants were married (89.4%), Han Chinese (97.2%), and well educated with college education or above (62.2%). Majority (41.1%) of the population were 25–35 years old and 45.6% were female.

**FIGURE 2:**
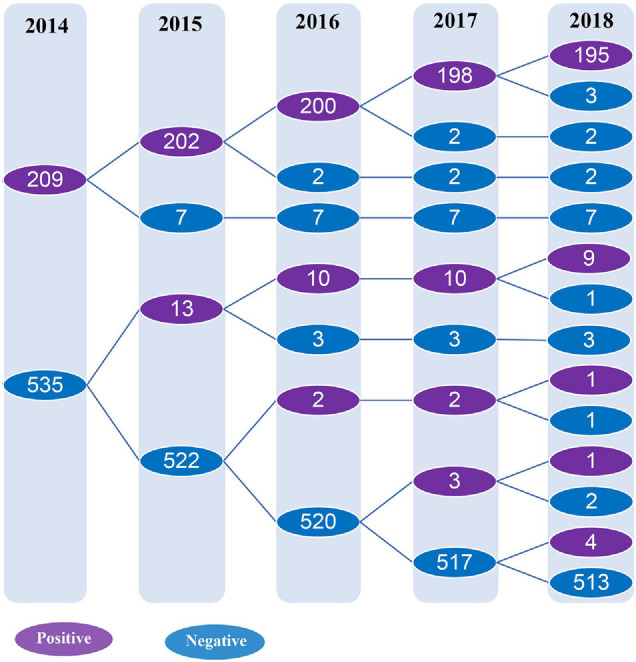
Detection of anti-*C. trachomatis* Pgp3 antibody from 2014 to 2018. The subjects included in the longitudinal cohort study were followed up and tested for anti-Pgp3 antibody annually. The purple circle indicates the number of anti-Pgp3 antibody positive subjects, while the blue circle represents the number of anti-Pgp3 antibody negative subjects.

Overall, 28.1% (209/744) serum samples tested positive for the antibody against *C. trachomatis* Pgp3 in 2014 while the frequencies of anti-Pgp3 from 2015 to 2018 was 28.9% (215/744), 28.5% (212/744), 28.6% (213/744), and 28.2% (210/744), respectively, with no statistical difference (*p* = 0.998, Figure 2).

**FIGURE 3:**
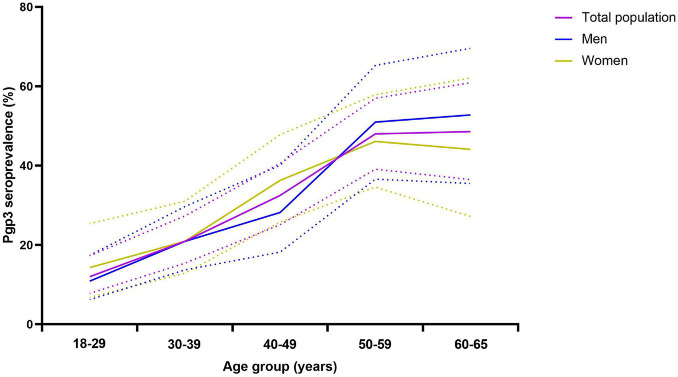
Frequency of anti-Pgp3 antibody by age groups in total population (purple), men (blue) or women (brown), China, 2014. Anti-Pgp3 antibody was tested in 744 participants including 405 men and 339 women in 2014. Seropositive frequency of *C. trachomatis* infection was represented by anti-Pgp3 IgG antibody positive rate. Solid lines indicate the frequency for whole population (purple), men (blue) or women (brown), respectively. Dash lines indicate the 95% “exact” Clopper–Pearson confidence interval of the seropositive frequency.

The CT seropositive frequency was significantly different among various age groups (*p* < 0.0001, Table 2) and rose when the ages increased. The lowest CT frequency (12.0%) was observed in the age group of 18–29 years while the frequency gradually increased up to 48.6% in the group of 60–65 years old. The pattern of CT infection was quite similar for men or women (Figure 3) although the CT seropositive frequency was slightly higher in women than in men (31.3% vs. 25.4%) but without statistical significance (Table 2).

**TABLE 2:** Frequency of antibody against *C. trachomatis* Pgp3 protein in a community population of Northern China in 2014.

Characteristics	Whole population		Men				Women		
			
	No.	Frequency %	*P* value^#^	No.	Frequency %	*P* value	No.	Frequency %	*P* value
	tested	(95% CI)^*^		tested	(95% CI)		tested	(95% CI)	
**Age group (years)**									
18–29	200	12.0 (7.8–17.3)	**<0.001**	137	10.9 (6.2–17.4)	**<0.001**	63	14.3 (6.8–25.4)	**<0.001**
30–39	196	20.9 (15.4–27.3)		110	20.9 (13.7–29.7)		86	20.9 (12.9–31.0)	
40–49	151	32.5 (25.1–40.6)		71	28.2 (18.2–40.1)		80	36.3 (25.8–47.8)	
50–59	127	48.0 (39.1–57.0)		51	51.0 (36.6–65.3)		76	46.1 (34.6–57.9)	
60–65	70	48.6 (36.5–60.9)		36	52.8 (35.5–69.6)		34	44.1 (27.2–62.1)	
Total	744	28.1 (24.9–31.5)		405	25.4 (17.3–34.9)		339	31.3 (26.4–36.5)	
**Ethnicity**									
Han	723	28.4 (25.1–31.8)	0.35	393	26 (21.7–30.6)	0.31	330	31.2 (22.4–41.1)	>0.999
Non-han	21	19.0 (5.4–41.9)		12	8.3 (0.21–38.4)		9	33.3 (7.5–70.0)	
**Marital status**									
Married	665	29.6 (26.2–33.2)	**0.005**	349	28.1 (23.4–33.1)	**0.006**	316	31.3 (26.2–36.7)	0.456
Unmarried	62	11.3 (4.7–21.9)		51	9.8 (3.3–21.4)		11	18.2 (2.3–51.8)	
Divorced/widowed	17	29.4 (10.3–56.0)		5	0 (0–52.2)		12	41.7 (15.2–72.4)	
**Education levels**									
College/above	463	21.8 (18.1–25.8)	**<0.001**	291	20.6 (16.1–25.7)	**0.001**	172	23.8 (17.7–30.9)	**0.01**
High school	162	37.0 (29.6–44.9)		68	32.4 (21.6–44.8)		94	40.4 (30.4–51.0)	
Junior school/below	119	40.3 (31.4–49.7)		46	45.7 (30.9–61.0)		73	37.0 (26.0–49.1)	

*95% “exact” Clopper-Pearson confidence interval of the frequency, estimated by the MedCalc software.

*^#^*P* values were calculated using chi-square tests with SPSS version 22.0 statistical software.*

*Bold values indicate statistically significant associations, with *P* < 0.05.*

Furthermore, CT infection was more frequently observed in those with lower education levels (38.4% vs. 21.8%, *p* < 0.001) and in the married persons (29.6% vs. 11.3%, *p* = 0.005, Table 2). However, multivariable Poisson regression analysis indicated that age was the only factor that was significantly associated with CT seropositivity (*p* < 0.001, Table 3). Further stratified analysis also confirmed that both marital status and education levels were the confounding factors due to the unbalanced age distribution across different marital status and education levels (Supplementary Table 2) and were not associated with the CT seropositivity (Supplementary Table 3).

**TABLE 3:** Factors associated with the detection of anti-*C. trachomatis* Pgp3 IgG in a community population of Northern China in 2014.

Characteristics	Whole population		Men		Women	
			
	FR* (95% CI)	Adj-FR^#^ (95% CI)	FR (95% CI)	Adj-FR (95% CI)	FR (95% CI)	Adj-FR (95% CI)
**Age group (years)**						
18–29	1^∮^	1	1	1	1	1
30–39	**1.74 (1.05–2.88)**	1.67 (0.96–2.9)	1.91 (0.99–3.66)	1.78 (0.86–3.68)	1.47 (0.66–3.26)	1.52 (0.65–3.56)
40–49	**2.72 (1.67–4.43)**	**2.63 (1.51–4.58)**	**2.61 (1.34–5.1)**	**2.42 (1.13–5.18)**	**2.54 (1.2–5.36)**	**2.65 (1.16–6.03)**
50–59	**4.00 (2.5–6.42)**	**3.95 (2.22–7.02)**	**4.66 (2.47–8.79)**	**4.74 (2.16–10.37)**	**3.22 (1.55–6.71)**	**3.43 (1.46–8.07)**
60–65	**4.05 (2.4–6.83)**	**4.13 (2.16–7.9)**	**4.69 (2.38–9.23)**	**4.94 (1.98–12.31)**	**3.09 (1.35–7.06)**	**3.48 (1.34–9.03)**
**Ethnicity**						
Han	1	1	1	1	1	1
Non-Han	0.67 (0.25–1.81)	0.88 (0.32–2.4)	0.32 (0.04–2.3)	0.42 (0.06–3.07)	1.07 (0.34–3.37)	1.44 (0.44–4.68)
**Marital status**						
Married	1	1	1	1	1	1
Unmarried	**0.38 (0.18–0.81)**	0.88 (0.37–2.07)	**0.35 (0.14–0.86)**	0.8 (0.28–2.27)	0.58 (0.14–2.35)	1.3 (0.28–5.96)
Divorced/widowed	0.99 (0.41–2.41)	0.82 (0.34–2.02)	–	–	1.33 (0.54–3.27)	0.99 (0.4–2.47)
**Education levels**						
College/above	1	1	1	1	1	1
High school	**1.7 (1.23–2.34)**	1.03 (0.71–1.48)	1.57 (0.96–2.56)	0.8 (0.45–1.41)	**1.7 (1.1–2.64)**	1.22 (0.75–1.99)
Junior school/below	**1.85 (1.31–2.61)**	0.87 (0.57–1.35)	**2.21 (1.34–3.64)**	0.88 (0.45–1.7)	1.55 (0.95–2.52)	0.89 (0.49–1.6)

*FR, frequency ratio, estimated by univariable Poisson regression.

*^#^Adj-FR, adjusted frequency ratio, estimated by multivariable Poisson regression.*

*^∮^ Reference, the group is set as control group; boldface indicates significant association, where the 95% confidence interval does not cross the null value of 1.0.*

### Incidence and Reinfection Rate of *C. trachomatis* Infection

During the 4 years of follow-up, 744 subjects contributed a total of 2,976 person-years of observation, including 2,094 person-years from CT-seronegative subjects and 882 person-years from CT seroconverted subjects. A total of 22 CT new infections and 12 reinfections were recorded. Therefore, the CT incidence and the reinfection rate were 11 per 1,000 person-years (95% CI, 7–17) and 14 per 1,000 person-years (95% CI, 7–24), respectively, during the period from 2014 to 2018 (Table 4). In terms of each observational year, the incidence in 2015 was significantly higher (24 per 1,000 person-years, 95% CI, 13–41) than in other years (*p* = 0.003, Table 4) whereas the reinfection rate was similar among the follow-up years (*p* = 0.673, Table 4). Furthermore, for both CT incidence and reinfection rate, no significant difference was observed with respect to age, gender, ethnicity, marital status, and education levels although relatively high incidence and reinfection rate of CT were observed in the age group of 18–29 (Table 4 and Supplementary Table 4).

**TABLE 4:** Incidence and reinfection rate of *C. trachomatis* infection in a community population of Northern China during 2014 and 2018.

Characteristics	Incidence*			Reinfection rate^#^				
		
	No. of person-years	No. new infection (per 1,000 person-years	95% CI^∮^ (per 1,000 person-years	*P* ^∫^ value	No. of person-year	No. reinfection (per 1,000 person-years)	95% CI (per 1,000 person-years)	*P* value
**Age group (years)**								
18–29	308	6 (19)	7–42	0.387	56	2 (36)	4–123	0.183
30–39	909	9 (10)	5–19		196	5 (26)	8–59	
40–49	386	5 (13)	4–30		196	2 (10)	1–36	
50–59	264	1 (4)	0.1–21		219	1 (5)	0.1–25	
60–70^ξ^	227	1 (4)	0.1–24		215	2 (9)	1–33	
Total	2094	22 (11)	7–17		882	12 (14)	7–24	
**Gender**								
Men	1179	13 (11)	6–19	0.833	441	7 (16)	6–33	0.561
Women	915	9 (10)	5–19		441	5 (11)	4–26	
**Race**								
Han	2027	21 (10)	6–15	0.513	865	12 (14)	7–24	>0.999
Non-Han	67	1 (15)	0–80		17	0	–	
**Marital status**								
Married	1837	16 (9)	5–15	0.059	823	11 (13)	7–24	0.567
Unmarried	212	5 (24)	8–55		36	1 (28)	1–145	
Divorced/widowed	45	1 (22)	1–117		23	0	–	
**Education levels**								
College/above	1413	17 (12)	7–19	0.52	439	7 (16)	6–33	0.932
High school	399	4 (10)	3–25		249	3 (12)	3–35	
Junior school/below	282	1 (4)	0–20		194	2 (10)	1–37	
**Follow-up year**								
2015	535	13 (24)	13–41	**0.003**	209	4 (19)	5–48	0.673
2016	522	2 (4)	1–14		222	2 (9)	1–32	
2017	520	3 (6)	1–17		224	4 (18)	5–45	
2018	517	4 (8)	2–20		227	2 (9)	1–31	

*The CT new infection was defined as seroconversion to anti-Pgp3 IgG from anti-Pgp3 negative subjects, the CT incidence is determined by dividing the number of CT new infected individuals with the person-years observed during the study period of 2014 and 2018.

*^#^The CT reinfection was determined when the titers of anti-Pgp3 IgG increased by at least fourfolds among the subjects positive for anti-Pgp3 IgG, the CT reinfection rate is calculated by dividing the number of CT reinfected individuals with the person-years observed.*

*^∮^ 95% “exact” Clopper-Pearson confidence interval.*

*^∫^*P* values were calculated using chi-square tests.*

*ξPerson-years observed in 65–70-year age group result from the follow-up of participants aged 61–65 years old.*

*Bold values indicate statistically significant associations, with *P* < 0.05.*

### Kinetics of Anti-*C. trachomatis* Pgp3 IgG Antibodies

A total of 209 subjects were initially found to be seropositive for anti-Pgp3 antibody at the beginning of the study in 2014 and 93.3% (195/209) of them remained seropositive at the end of our study in 2018 (Figure 2), indicating a seroreversion rate of 6.7% (14/209) in this population during the 4 years analyzed. From 2015 to 2018, the annual seroreversion rate of anti-Pgp3 antibody was 3.3% (7/209), 1.0% (2/202), 1.0% (2/200), and 1.5% (3/198), respectively, with no significant difference.

Luciferase immunosorbent assay is a semi-quantitative assay and could assess the titers and the dynamics of anti-Pgp3 antibody in this longitudinal cohort. In 2014, 209 seropositive subjects showed an average titer of 7.72 Log2 RLU (95% CI, 7.33–7.90) for anti-Pgp3 antibody. According to the titers of anti-Pgp3 antibody in 2014, CT-positive subjects were then divided into three groups, i.e., strong (top 25%), moderate (25–75%), and weak (lower 25%), respectively (Table 5). As we can see, no significant difference was observed among the three groups with respect to gender, ethnicity, marital status, and education levels (Table 5). The subjects with high antibody titers were relatively younger than those in the moderate group (44 vs. 49 years old, *p* = 0.019, Table 5). Notably, the positive rate of anti-Pgp3 IgG3 antibody, a biomarker of recent CT infection, was significantly different among the three groups, i.e., 11.5, 29.8, and 66% for weak, moderate, and strong antibody response, respectively (*p* < 0.001, Table 5).

**TABLE 5:** Characteristics of *C. trachomatis* seropositive subjects with different titers of anti-Pgp3 IgG antibody.

	Anti-*C. trachomatis* Pgp3 IgG				
		
	Total (*N* = 209)	Weakly positive*	Moderately positive	Strongly positive	*P*^#^ value
		*N* = 52)*	(*N* = 104)	(*N* = 53)	
**Baseline characteristics**					
Anti-Pgp3 IgG antibody Mean,95% CI (Log2 RLU)	7.62 (7.33–7.90)	5.1 (4.9–5.3)	7.4 (7.2–7.6)	10.5 (10.2–10.8)	**<0.001**
Age (years) (median, interquartile range)	47 (36–57)	49 (33–58)	**49 (41–59)**	**44 (32–51)**	**0.019**
Gender (n, %)					
Male	103 (49.3)	26 (50.0)	49 (47.1)	28 (52.8)	0.789
Female	106 (50.7)	26 (50.0)	55 (52.9)	25 (47.2)	
Ethnicity (n, %)					
Han	205 (98.1)	51 (98.1)	103 (99.0)	51 (96.2)	0.561
Non-Han	4 (1.9)	1 (1.9)	1 (1.0)	2 (3.8)	
Marital status (n, %)					
Unmarried	7 (3.3)	3 (5.8)	1 (1.0)	3 (5.7)	0.224
Married	197 (94.3)	47 (90.4)	101 (97.1)	49 (92.5)	
Divorced/widowed	5 (2.4)	2 (3.8)	2 (1.9)	1 (1.9)	
Education levels (n, %)					
Junior school/below	48 (23.0)	13 (25.0)	28 (26.9)	7 (13.2)	0.378
High school	60 (28.7)	13 (25.0)	29 (27.9)	18 (34.0)	
College/above	101 (48.3)	26 (50.0)	47 (45.2)	28 (52.8)	
IgG3 of anti-Pgp3 (n, %)					
Positive	72 (34.4)	**6 (11.5)**	**31 (29.8)**	**35 (66.0)**	**<0.001**
Negative	137 (65.6)	46 (88.5)	73 (70.2)	18 (34.0)	
Seroreversion of anti-Pgp3 (n, %)	14 (6.7)	12 (23.1)	1 (1.0)	1 (1.9)	**<0.001**
Average decay rate (Log2 RLU/year, 95% CI)	−0.123 (−0.155, −0.091)	−0.146 (−0.229, −0.063)**^*a*^**	−0.076 (−0.093, −0.059)**^*b*^**	−0.186 (−0.269, −0.103)**^*c*^**	*P*_*ab*_ = **0.018** *P*_*bc*_ = **0.005** *P*_*ac*_ = 0.296

*According to the results of 2014, 209 initial seropositive subjects were divided into three groups based on the rank of anti-Pgp3 antibody concentration, strongly positive (top 25%), moderately positive (25–75%) and weakly positive (lower 25%).

*^#^*P* value was calculated using chi-square tests (Pearson chi-square, continuity correction, and Fisher’s exact test) for categorical variables and Mann–Whitney *U*-test for non-categorical variables in SPSS. *P*_*ab*_, *P*_*bc*_, and *P*_*ac*_ were empirical *P* values, calculating by the Fisher’s permutation test in Stata.*

*^∮^ Of 209 initial positive subjects, 198 were included for the analysis of anti-Pgp3 IgG dynamics while 11 were excluded due to the CT reinfection during the later follow-up.*

*Decay rate of anti-Pgp3 IgG antibody was estimated using Generalized Estimating Equation model implemented in Stata.*

*Superscript “a” represents the average decay rate of a weakly positive group, whereas superscripts “b” and “c” represent the decay rates of a moderately positive group and a strongly positive group, respectively. *P*_*ab*_ indicates the *P*-value for the comparison of average decay rate between a weakly positive group and a moderately positive group, similarly for *P*_*bc*_ and *P*_*ac*_.*

*Bold values indicate statistically significant associations, with *P* < 0.05.*

Next, we adapted GEE analysis to estimate the antibody decay rate for anti-Pgp3 over years. A total of 198 out of 209 CT seropositive subjects identified in 2014 were included for the longitudinal antibody analysis, while another 11 seropositive subjects were excluded due to increased titers of anti-Pgp3 antibody caused by CT reinfection during the follow-up. In general, the titers of anti-Pgp3 IgG antibody gradually declined over the years with a slope of −0.123 Log2 RLU/year (95% CI: −0.155 to −0.091) with slight difference of anti-Pgp3 decay rate among the three groups with different antibody titers (Table 5).

**FIGURE 4:**
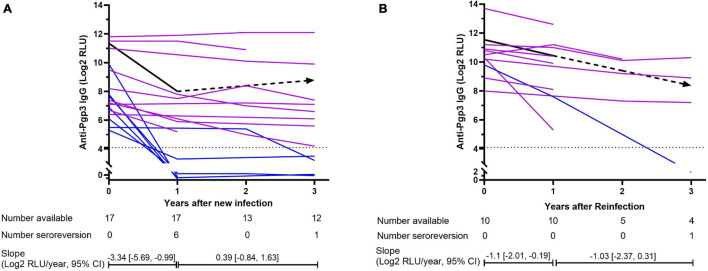
Dynamics of anti-Pgp3 antibody in *C. trachomatis* new infection **(A)** and reinfection **(B)**. Dynamics of anti-Pgp3 antibody was analyzed in 17 newly CT-infected subjects and 10 CT reinfected subjects. Purple lines indicate the subjects who were seropositive for anti-Pgp3 during the follow-up, whereas blue lines indicate those with seroreversion. Black line represents the dynamics of antibody change estimated by GEE model analysis in which the solid lines mean the trend of antibody change is statistically significant while dash lines mean no statistically significant. In addition, dot lines indicate the cutoff value of anti-Pgp3 IgG antibody (Log2 RLU = 4.1). Slopes were estimated by GEE models.

However, the newly CT-infected subjects showed significantly lower titers of anti-Pgp3 antibody than CT-reinfected subjects (7.65 vs. 10.35 Log2 RLU, *p* < 0.001) when CT new infection or reinfection was diagnosed, and relatively high frequency (41.2%, 7/17) of seroreversion of anti-Pgp3 antibody during the median follow-up period of 3 years. Of note, 85.7% (6/7) of these seroreversions occurred within the first year after CT new infection (Figure 4A). In contrast, CT reinfected-individuals experienced much lower seroreversion rate (10%, 1/10, Figure 4B) during the median follow-up period of 1.5 years (*p* < 0.001). Furthermore, GEE analysis confirmed the rapid decay of anti-Pgp3 antibody titers over time in particular in the first year after CT infection or reinfection with the decay rate of 3.34 Log2 RLU/year for CT new infection and 1.1 Log2 RLU/year for CT reinfection, respectively (Figure 4).

## Discussion

To the best of our knowledge, this is the first longitudinal study about the seroepidemiology of *C. trachomatis* infection in the general population of China. We found that at least one quarter of the people aged 18–65 years have been infected with *C. trachomatis* over their lifetime based on the frequency of anti-Pgp3 antibody, while all age groups are susceptible to *C. trachomatis* infection, although the incidence and reinfection rate are relatively low (about 10 per 1,000 person-year) in the community of Northern China.

The most significant advantage of seroepidemiologic analysis is its capability to reflect both current and past *C. trachomatis* infection. Although there are no serologic assays approved by the US Food and Drug Administration (FDA), the preliminary evaluation of our in-house LISA by using sera from *C. trachomatis* NAAT-positive CT-infected women and low-risk children showed high sensitivity and specificity, which are comparable with the commercial Mikrogen ELISA. Although our “in-house” LISA used one Pgp3 protein from serovar E of *C. trachomatis*, it does show comparable performance with the commercial Mikrogen ELISA, which uses multiple *C. trachomatis* antigens (MOMP, TARP, and CPAF). Of note, Jones et al. (2020) recently reported the variation of Pgp3 protein with a genetic distance of 0.013 at the amino acid level among different *C. trachomatis* serovars. However, the small genetic distance of Pgp3 gene and the corresponding 98.7% sequence identity maintains a 94% probability of cross-reactivity within *C. trachomatis* species according to the formula


``P=cross e/(-9.4153+ 0.123223×percentsequenceidentity)[1+e](-9.4153+0.123223×percentsequenceidentity)″

reported by Rahman et al. (2015, 2018b). In our study, we also validate the cross-reactivity of Pgp3 protein among different serovars analyzed. Eight NAAT-positive women (6.4%), including one infected with serovar E and seven with unknown serovars, were negative for both Pgp3-based LISA and Mikrogen ELISA. The lack of CT-specific antibody response has been previously reported in 5.5–10.2% of CT-NAAT positive subjects (Wang and Grayston, 1974; Komoda, 2007; Geisler et al., 2013; Bakshi et al., 2017) due to early CT infection without antibody response, reduced antigenic burden resulting from early therapy, inadequate dose of *C. trachomatis* to cause productive infection, or immunosuppressive diseases to prevent the production of CT specific antibodies (Geisler et al., 2012). However, in our study, the CT-NAAT-positive but antibody-negative patient was most likely in the early stage of CT infection since we found that the patient was negative for IgG antibody against Pgp3, but positive for IgM antibody against Pgp3. Our preliminary data supported the “in-house” Pgp3-based LISA as a suitable assay for the detection of anti-*C. trachomatis* antibody.

In our study, the overall seropositive frequency of *C. trachomatis* infection was 28.1% for the people aged 18–65 years old at baseline. To compare our results with previous studies, we adjusted the *C. trachomatis* seropositive frequency, incidence, and reinfection rate according to the assay performance, age groups, and gender within the study populations (Supplementary Table 5). The adjusted CT seropositive frequency in our study was similar to that in non-STD clinic outpatients from Tianjin, China (17.6% vs. 22.9%, *p* = 0.077) (Zhang et al., 2016), but significantly lower than that in the British general population (17.9% vs. 30.4% in men, *p* = 0.001 and 22.5% vs. 34.8% in women, *p* = 0.008) (Woodhall et al., 2017) and in the females from the general population of the United States (19.5% vs. 34.4%, *p* = 0.004) (Petersen et al., 2020). However, the adjusted frequency in our study was still relatively higher than that in the general population of Netherlands, especially more significant in men (16.6% vs. 8.1%, *p* = 0.004) (van Aar et al., 2014). Our results indicated that as the most common STI, exposure to *C. trachomatis* at some point over the lifetime is quite common in Northern China and could be as high as 50% in the sub-population aged 60–65 years.

Furthermore, our study uncovered the *C. trachomatis* incidence of 11 per 1,000 person-years in China based on the detection of anti-Pgp3 antibody seroconversion and the *C. trachomatis* reinfection rate of 14 per 1,000 person-years based on the fourfold increase of anti-Pgp3 antibody titers. The adjusted incidence was similar to that observed in the general population of New Zealand (14 per 1,000 vs. 19 per 1,000 person-year, *p* = 0.326) based on anti-Pgp3 seroconversion (Righarts et al., 2017) but significantly lower than that obtained in the community population of Australia (55 per 1,000 person-year, *p* < 0.001) based on the NAAT (Silver et al., 2015). The adjusted reinfection rate (18 per 1,000 person-years) in our study was similar to that in the American soldiers aged 16–51 years (21 per 1,000 person-year, *p* = 0.655) based on NAAT (Barnett and Brundage, 2001). Of note, the CT incidence identified in our study was 20–30-fold higher than that (0.37–0.55 per 1,000 person-year) reported from the national STI surveillance in China from 2015 to 2019 (Yue et al., 2020), suggesting a serious underestimation of *C. trachomatis* new infection rate in China partly due to 80% asymptomatic CT infection and the large number of undiagnosed CT infection as well as low reporting rate of the current surveillance system.

Like other STIs, there is a trend of age-related increasing seropositive frequency of *C. trachomatis* infection in the general population (van Aar et al., 2014; Woodhall et al., 2017). Previous studies found that CT seropositivity usually reached the peak at the age of 30–35 years followed by a slight decrease in older age groups (Woodhall et al., 2017). In our study, the CT seropositive frequency increased over ages, suggesting a broad susceptibility of all age groups to *C. trachomatis* infection in China. The susceptibility of all age groups to *C. trachomatis* infection was also supported by the similar CT incidence among different age groups in our study. In general, *C. trachomatis* incident cases (Scott Lamontagne et al., 2007; Walker et al., 2012; Silver et al., 2015) and NAAT-positive detections (Marcone et al., 2012; Sonnenberg et al., 2013) are more often found in teenagers (15–19 years) and declined with age thereafter. Two studies based on seroconversions also reported that the highest incidence occurred in those under 26 years in New Zealand (Righarts et al., 2017) and pregnant women under 23 years in Finland (Lyytikäinen et al., 2008b). These results are in line with the recommendation of the annual STI screening for sexually active women under the age of 25 in the United States [Centers for Disease Control and Prevention (CDC), 2019] and European countries [European Centre for Disease Prevention and Control (ECDC), 2008]. However, in our study, we failed to see the significantly higher incidence of CT infection around the age of 25 years, although STI surveillance data showed a 25-year-old population as a high risk of CT infection in China (Yue et al., 2020).

The results about the association of CT infection and genders are inconsistent. Several studies found lower CT seropositive frequency in men than in women and no decrease of *C. trachomatis* frequency over ages in men (van Aar et al., 2014; Horner et al., 2016; Woodhall et al., 2017). The lower CT seropositive frequency in men may be due to lower antibody-inducing infection rate (Närvänen et al., 1997), weaker antibody response post-infection (Bessho and Matsumoto, 1990), and the sub-performance of serological assays in men (Wills et al., 2009). However, in our study, slightly lower seropositive frequency was observed in men than in women (25.4% vs. 31.3%), but the difference was not statistically significant (*p* = 0.078). In addition, the *C. trachomatis* incidence was also similar in men and women (11 per 1,000 vs. 10 per 1,000 person-year, *p* = 0.833) in our study. Our results were in line with other studies based on anti-Pgp3 seroconversions or NAAT testing (Kim et al., 2011; de Walque et al., 2012; Sonnenberg et al., 2013; Rutherford et al., 2014; Corsenac et al., 2015; Silver et al., 2015; Ikonomidis et al., 2016; Matteelli et al., 2016; Babinská et al., 2017; Righarts et al., 2017; Huneeus et al., 2018), indicating no gender-related significant difference of CT infection.

*Chlamydia trachomatis* incidence and reinfection are important for CT prevention. Two studies in England and Australia reported a remarkably higher reinfection rate than incidence (22.3–29.9 per 100 person-year vs. 4.9–6.4 per 100 person-year in England; 22.3 per 100 person-year vs. 4.4 per 100 person-year in Australia.) in women aged 16–25 years (Scott Lamontagne et al., 2007; Walker et al., 2012). These results indicated the susceptibility of at least part (20–30%) of CT-infected subjects to the reinfection of *C. trachomatis* or the insufficient protection of CT-related immunity to *C. trachomatis* infection. A possible explanation is the “immunity arrest” effect of the timely antibiotic treatment (Vickers and Osgood, 2014) since nearly 20% untreated *C. trachomatis* infections can resolve spontaneously and the protective immunity in “self-cure” individuals has been previously reported (Geisler et al., 2013). However, quite similar *C. trachomatis* incidence (11 per 1,000 person-year) and reinfection rate (14 per 1,000 person-year) were found in the community population of northern China in our study. Previous studies found that for *C. trachomatis* new infection, the subjects who have been infected with CT experienced a nearly fivefold high risk than those who have never been infected (Scott Lamontagne et al., 2007; Walker et al., 2012); however, no difference of *C. trachomatis* incidence and reinfection rate were observed in our study, which may indicate the protective immunity after *C. trachomatis* infection or the lack of treatment-related “immunity arrest” in China.

Our study showed relatively a low seroreversion rate (6.7%) of the anti-Pgp3 antibody during the 4 years of follow-up. This finding was in agreement with a previous study in which 96.5% of CT seropositive women and 83.9% of men maintained seropositivity for 12 years (Horner et al., 2016). We also found that the titers of the anti-Pgp3 antibody remain stable with a half-life time of around 8 years. A similar decay rate of anti-*C. trachomatis* IgG antibody has been described by Puolakkainen et al. (1986) who reported that anti-*C. trachomatis* EB antibodies remain stable in 3–6 years. Interestingly, for the CT incident samples, we observed a rapid antibody reduction and a high seroreversion rate of 35.3% within the first year post-infection. Workowski et al. (1993) also reported a seroreversion rate of 31.6% within 5 months after *C. trachomatis* NAAT-positive test. Öhman et al. (2020) found that anti-*C. trachomatis* MOMP IgG antibodies were only detected in 65.5% of women within 3 months of CT diagnosis. However, different from our results, several studies reported a stable IgG level for at least 6 months (Cunningham, 1995; Geisler et al., 2012) or very low seroreversion rate (1.4–6%) within 1 year (Komoda, 2007; Bakshi et al., 2017) after a positive *C. trachomatis* NAAT test. Therefore, the dynamics of CT-specific IgG antibodies after *C. trachomatis* new infection needs to be further investigated. We believe that for the CT reinfection, pre-existent *C. trachomatis* antibody may be an important factor to affect the antibody dynamics since the reinfected individuals experienced a much lower seroreversion rate (10%) in our study. Therefore, we can further infer that slow anti-CT antibody decay may be a proxy of *C. trachomatis* reinfection, which can only be identified by serological assays because the NAAT test can only identify current *C. trachomatis* infection, but cannot distinguish primary infection from reinfection of *C. trachomatis.*

There are several limitations in our study. First, *C. trachomatis* infection refers to CT-specific antibody-inducing infection in our study. However, a lack of CT-specific antibody response has been reported in 5.5–10.2% of *C. trachomatis*-infected subjects (Wang and Grayston, 1974; Komoda, 2007; Geisler et al., 2012; Bakshi et al., 2017). Considering seroreversion over time, especially the rapid decay of CT-specific antibody in *C. trachomatis* newly infected individuals, serological investigation could underestimate the real burden of *C. trachomatis* infection. Second, lack of data about the STI history of participants as well as personal behavior and sex attitudes prevented further analysis of the risk factors associated with *C. trachomatis* infection. Third, previous studies in Europe and the United States indicate the 25-year-old females as a high-risk population of *C. trachomatis* infection. However, only 4% of the participants were 18–25 years old in our study, and no conclusion can be made about their risk or burden of *C. trachomatis* infection in our study. Finally, although there were no significant differences observed between the subjects included and excluded in this study with respect to age, gender, ethnicity, marital status, and education levels, we cannot ensure that the characteristics, such as participants’ STI history, personal behavior, and sex attitudes, were equal between these two groups due to the non-random sampling strategy. The 744 people with longitudinal records and serum samples may not represent the underlying population of the community. Therefore, we must be cautious in interpreting the current results, and further study is needed. Additionally, the community we investigated is special since the participants are all oil field employees and their family members. The results obtained in this community cannot be expanded to the entire Chinese population, although our study provided the first community-based seroepidemiology and antibody dynamics of *C. trachomatis* in China.

## Conclusion

In this community-based longitudinal cohort study, we presented an overall CT seropositive frequency of 28.1% for Chinese adults and up to 48.6% frequency for a 60–65-year-old population. Although CT incidence and reinfection rate were low, all age groups are susceptible to CT infection. Comprehensive *C. trachomatis* prevention strategies are thus needed, especially for the prevention of adverse reproductive outcomes in women.

## Data Availability Statement

The dataset presented in this study can be found in online repository at https://www.ncbi.nlm.nih.gov/genbank/ with an accession number of OK148602.

## Ethics Statement

The studies involving human participants were reviewed and approved by the Ethical Committee of the Jidong Oil-field Hospital of China National Petroleum Corporation. The patients/participants provided their written informed consent to participate in this study.

## Author Contributions

ST and YD conceived and designed the experiments. DX, JS, and CA collected the clinical sample and data. JS, JZ, HL, and YL performed the experiments. JS, DX, YD, and ST interpreted the results. JS made the tables and figures. JS and ST wrote the manuscript. All authors reviewed, revised, and approved the final report.

## Conflict of Interest

The authors declare that the research was conducted in the absence of any commercial or financial relationships that could be construed as a potential conflict of interest.

## Publisher’s Note

All claims expressed in this article are solely those of the authors and do not necessarily represent those of their affiliated organizations, or those of the publisher, the editors and the reviewers. Any product that may be evaluated in this article, or claim that may be made by its manufacturer, is not guaranteed or endorsed by the publisher.

## Footnotes

1
https://www.ecdc.europa.eu/en/publications-data
