# Supplementary material for: Seroepidemiology of Chlamydia trachomatis Infection in the General Population of Northern China: The Jidong Community Cohort Study
Source: Front Microbiol. 2021 Sep 27;12:729016. doi: 10.3389/fmicb.2021.729016 (PMC8507574; doi:10.3389/fmicb.2021.729016)
Supplement: Supplementary file 1 [file Data_Sheet_1.docx]

**Supplementary Material**

Table S1 Comparison of baseline characteristics between the subjects aged 18-65 years included and excluded in this study

| **Characteristics** | **Subjects Included N=744** | **Subjects Excluded N=7897** | **P value ^a^** |
| --- | --- | --- | --- |
| **Age group (years) (n, %)** | | | |
| 18-25 | 32 (4.3) | 382 (4.8) | 0.09 |
| 25-35 | 306 (41.1) | 2900 (36.7) |  |
| 35-45 | 138 (18.5) | 1731 (21.9) |  |
| 45-55 | 135 (18.1) | 1515 (19.2) |  |
| 55-65 | 133 (17.9) | 1369 (17.3) |  |
| **Gender (n, %)** | | | |
| Male | 405 (54.4) | 4121 (52.2) | 0.24 |
| Female | 339 (45.6) | 3776 (47.8) |  |
| **Ethnicity (n, %)** | | | |
| Han | 723 (97.2) | 7666 (97.1) | 0.874 |
| Non-Han | 21 (2.8) | 231 (2.9) |  |
| **Marital status (n, %)** | | | |
| unmarried | 62 (8.3) | 636 (8.1) | 0.529 |
| married | 665 (89.4) | 7124 (90.2) |  |
| Divorced/widowed | 17 (2.3) | 137 (1.7) |  |
| **Education levels (n, %)** | | | |
| Junior school / below | 119 (16.0) | 1213 (15.4) | 0.896 |
| High school | 162 (21.8) | 1744 (22.1) |  |
| University / above | 463 (62.2) | 4940 (62.6) |  |

.

a: *P* value was calculated using Chi-square test.

Table S2 Age difference for the subjects according to the characteristics analyzed

| **Characteristics** | **No. Tested** | **Age (year) ^a^** | | **P value ^b^** |
| --- | --- | --- | --- | --- |
|  |  | **Median** | **interquartile range** |  |
| **Gender** | | | | |
| Male | 405 | 34 | 28 - 47 | <0.001 |
| Female | 339 | 42 | 31 - 54 |  |
| **Ethnicity** | | | | |
| Han | 723 | 38 | 29 - 50 | 0.015 |
| Non-Han | 21 | 29 | 27 - 40 |  |
| **Marital status** | | | | |
| unmarried | 62 | 26 | 24 - 28 | <0.001 |
| married | 665 | 40 | 31 - 51 |  |
| Divorced/widowed | 17 | 49 | 33 - 55 |  |
| **Education levels** | | | | |
| Junior school / below | 119 | 59 | 50 - 61 | <0.001 |
| High school | 162 | 47 | 37 - 56 |  |
| University / above | 463 | 31 | 28 - 40 |  |

a: Enrolment age of participants.

b: *P* value was calculated by the Mann-Whitney U-test.

Table S3 Association between *C. trachomatis* seropositive frequency and marital status and education levels in a community population of Northern China in 2014

| **Age group (years)** | **Frequency (n/N, %)** | | | **Frequency (n/N, %)** | | |
| --- | --- | --- | --- | --- | --- | --- |
|  | **Unmarried** | **Married** | **P value** | **High school and below ^b^** | **College and above** | **P value ^c^** |
| 18-29 | 7/59 (11.9) | 17/140 (12.1) | 0.956 | 2/19 (10.5) | 22/181 (12.2) | 0.835 |
| 30-39 | 0/3 (0) | 40/188 (21.3) | 0.604 | 7/35 (20) | 34/161 (21.1) | 0.883 |
| 40-49 | - | 47/147 (32.0) | - | 20/65 (30.8) | 29/86 (33.7) | 0.701 |
| 50-59 | - | 59/121 (48.8) | - | 48/98 (49) | 13/29 (44.8) | 0.694 |
| 60-65 | - | 34/69 (49.3) | - | 31/64 (48.4) | 3/6 (50) | 0.942 |
| Total | 7/62 (11.3) | 197/665 (29.6) | 0.002 | 108/281 (38.4) | 101/463 (21.8) | <0.001 |

Table S4 Comparison of *C. trachomatis* incidence and reinfection rate in different years and characteristic groups during 2014-2018

| **Characteristics** | **Seroincidence (/1000 person-years)** | **Reinfection rate (/1000 person-years)** | **P value ^a^** |
| --- | --- | --- | --- |
| **Age group (years)** | |  |  |
| 18-29 | 19 | 36 | 0.355 |
| 30-39 | 10 | 26 | 0.156 |
| 40-49 | 13 | 10 | >0.999 |
| 50-59 | 4 | 5 | >0.999 |
| 60-70 | 4 | 9 | 0.962 |
| Total | 11 | 14 | 0.468 |
| **Gender** | |  |  |
| Male | 11 | 16 | 0.432 |
| Female | 10 | 11 | >0.999 |
| **Ethnicity** | |  |  |
| Han | 10 | 14 | 0.415 |
| Non-Han | 15 | 0 | >0.999 |
| **Marital status** | |  |  |
| unmarried | 24 | 28 | >0.999 |
| married | 9 | 13 | 0.268 |
| Divorced/widowed | 22 | 0 | >0.999 |
| **Education levels** | |  |  |
| Junior school / below | 4 | 10 | 0.744 |
| High school | 10 | 12 | >0.999 |
| University / above | 12 | 16 | 0.526 |
| **Year of investigation** | |  |  |
| 2015 | 24 | 19 | 0.88 |
| 2016 | 4 | 9 | 0.737 |
| 2017 | 6 | 18 | 0.249 |
| 2018 | 8 | 9 | >0.999 |

a: *P* value was calculated using Chi-square tests.

Table S5 Summary of *C. trachomatis* seroprevalence, incidence and reinfection rate in previous studies

1. Seroprevalence of *C. trachomatis* infection.

| **Study** | **Country and city** | **Date of study** | **Subjects** | **Antibody tested** | **Whole population** | | | | **Men** | | | | **Women** | | | |
| --- | --- | --- | --- | --- | --- | --- | --- | --- | --- | --- | --- | --- | --- | --- | --- | --- |
|  |  |  |  |  | **Sp %**  **^a^** | **Adj-Sp  % ^b^** | **Adj-Sp %  (our study) ^c^** | **P ^d^  value** | **Sp %** | **Adj-Sp  %** | **Adj-Sp %  (our study)** | **P  value** | **Sp %** | **Adj-Sp  %** | **Adj-Sp %  (our study)** | **P  value** |
| Zhang XM et al.  2016 [37] | China, Tianjin | 2014.3 - 2014. 12 | Non-STD clinic outpatients, 15 - 40 years | Combination of Pgp3, CT875, CT694 | 21.0 | 22.9 | 17.6 | 0.077 | NA | NA | NA | NA | NA | NA | NA | NA |
| Woodhall et al. 2017 [25] | England | 1994 - 2012 | General population in the nationally-representative Healthy Surveys, 16 - 44 years | Pgp3 | 19.7 | 32.2 | 19.7 | **<0.001** | 13.9 | 30.4 | 17.9 | **0.001** | 24.4 | 34.8 | 22.5 | **0.008** |
| van Aar et al.  2014 [28] | Netherlands | 1996 and 2007 | General population,  15 - 39 years | MOMP | 7.7 | 12.2 | 17.7 | **0.028** | 5.6 | 8.1 | 16.6 | **0.004** | 9.8 | 16.1 | 19.5 | 0.449 |
| Petersen et al.  2020 [7] | United States of America | 2013 - 2016 | Women from national health and nutrition examination surveys, 18 - 39 years | Pgp3 | NA | NA | NA | NA | NA | NA | NA | NA | 30 | 34.4 | 19.5 | **0.004** |

1. Incidence of *C. trachomatis* infection

| **Study** | **Country and city** | **Date of study** | **Subjects** | **Testing methods** | **Total population (/1000 py) ^e^** | | | | **Men (/1000 py)** | | | | **Women (/1000 py)** | | | |
| --- | --- | --- | --- | --- | --- | --- | --- | --- | --- | --- | --- | --- | --- | --- | --- | --- |
|  |  |  |  |  | **Inci** | **adj-Inci** | **adj-Inci  (our study)** | **P  value** | **Inci** | **adj-Inci** | **adj-Inci  (our study)** | **P  value** | **Inci** | **adj-Inci** | **adj-Inci  (our study)** | **P  value** |
| Righarts AA et al. 2017 [26] | New Zealand | 1998 - 2011 | General population from a birth cohort, under 38 years | Anti-Pgp3 IgG | 16 | 19 | 14 | 0.326 | 13 | 22 | 14 | 0.332 | 19 | 15 | 13 | 0.732 |
| Silver BJ et al. 2015 [38] | Australia | 2009 - 2011 | Adolescents and young adults from communities, over 16 years | Nucleic acid test | 95 | 55 | 13 | **<0.001** | 86 | 57 | 13 | **<0.001** | 100 | 53 | 13 | **0.001** |

1. Reinfection rate of *C. trachomatis* infection.

| **Study** | **Country and city** | **Date of study** | **Population and age, years** | **Testing methods** | **Total population (/1000 py)** | | | |
| --- | --- | --- | --- | --- | --- | --- | --- | --- |
|  |  |  |  |  | **Reinfection rate** | **adj-Reinfection rate** | **adj-Reinfection rate  (our study)** | **P  value** |
| Barnett SD et al. 2001 [39] | USA | 1994 - 1998 | Soldiers with CT infection, 16 - 51 years | Nucleic acid test | 52 | 21 | 18 | 0.655 |

a: Sp, seroprevalence, directly reported by previous studies, so do the Inci (incidence) and reinfection rate;

b: Adj-Sp, adjusted-seroprevalence for previous study, the reported seroprevalence adjusted by their assays firstly, and then adjusted according to the age-gender distribution in our study for the total population, according to the age distribution in our study for men or women. So do the adj-Inci (adjusted-incidence) and adj-Reinfection rate;

c: Adjusted-seroprevalence for our study, the seroprevalence is adjusted by the sensitivity (92.8%) and specificity (100%) of our serological assay. So do the adj-Inci (adjusted-incidence) and adj-Reinfection rate for our study;

d: *P* value was calculated using Chi-square tests.

e: /1000 py, per 1000 person-year.

**Fig S1 Anti-Pgp3 IgG responses according to different *C. trachomatis* serovars.**
